# Sex specific function of epithelial STAT3 signaling in pathogenesis of *K-ras* mutant lung cancer

**DOI:** 10.1038/s41467-018-07042-y

**Published:** 2018-11-02

**Authors:** Mauricio S. Caetano, Maya Hassane, Hieu T. Van, Emmanuel Bugarin, Amber M. Cumpian, Christina L. McDowell, Carolina Gonzalez Cavazos, Huiyuan Zhang, Shanshan Deng, Lixia Diao, Jing Wang, Scott E. Evans, Carmen Behrens, Ignacio I. Wistuba, Susan A. W. Fuqua, Huang Lin, Laura P. Stabile, Stephanie S. Watowich, Humam Kadara, Seyed Javad Moghaddam

**Affiliations:** 1 Department of Pulmonary Medicine, The University of Texas MD Anderson Cancer Center, Houston, 77030 TX USA; 2 Department of Biochemistry and Molecular Genetics, Faculty of Medicine, American University of Beirut, Beirut, 11072020 Lebanon; 3 Tecnológico de Monterrey, Escuela de Medicina y Ciencias de la Salud, Monterrey, 64710 Nuevo León Mexico; 4 Department of Translational Molecular Pathology, The University of Texas MD Anderson Cancer Center, Houston, 77030 TX USA; 5 Department of Immunology, The University of Texas MD Anderson Cancer Center, Houston, 77030 TX USA; 6 Department of Respiratory Medicine, Second Affiliated Hospital, Xi’an Jiaotong University, Xi’an, 710004 China; 7 Department of Bioinformatics & Computational Biology, The University of Texas MD Anderson Cancer Center, Houston, 77030 TX USA; 8 Department of Thoracic/Head and Neck Medical Oncology, The University of Texas MD Anderson Cancer Center, Houston, 77030 TX USA; 9 Lester and Sue Smith Breast Center, Baylor College of Medicine, Houston, 77030 TX USA; 10 Department of Biostatistics, UPMC Hillman Cancer Center, University of Pittsburgh, Pittsburgh, 15232 PA USA; 11 Department of Pharmacology & Chemical Biology, UPMC Hillman Cancer Center, University of Pittsburgh, Pittsburgh, 15232 PA USA; 12 Department of Epidemiology, The University of Texas MD Anderson Cancer Center, Houston, 77030 TX USA; 13 The University of Texas M.D. Anderson Cancer Center UTHealth Graduate School of Biomedical Sciences, Houston, 77030 TX USA

## Abstract

Lung adenocarcinomas (LUADs) with mutations in the *K-ras* oncogene display dismal prognosis. Proinflammatory and immunomodulatory events that drive development of *K-ras* mutant LUAD are poorly understood. Here, we develop a lung epithelial specific *K-ras* mutant/*Stat3* conditional knockout (LR/*Stat3*^Δ/Δ^) mouse model. Epithelial *Stat3* deletion results in intriguing sex-associated discrepancies; *K-ras* mutant tumors are decreased in female LR/*Stat3*^Δ/Δ^ mice whereas tumor burdens are increased in males. RNA-sequencing and tumor microenvironment (TME) analysis demonstrate increased anti-tumor immune responses following *Stat3* deletion in females and, conversely, elevated pro-tumor immune pathways in males. While IL-6 blockade in male LR/*Stat3*^Δ/Δ^ mice reduces lung tumorigenesis, inhibition of estrogen receptor signaling in female mice augments *K-ras* mutant oncogenesis and reprograms lung TME toward a pro-tumor phenotype. Our data underscore a critical sex-specific role for epithelial *Stat3* signaling in *K-ras* mutant LUAD, thus paving the way for developing personalized (e.g. sex-based) immunotherapeutic strategies for this fatal disease.

## Abstract (web summary)

Proinflammatory and immunomodulatory events that drive development of *K-ras* mutant lung adenocarcinoma (LUAD) are poorly understood. Here they develop a lung epithelial specific *K-ras* mutant/*Stat3* conditional knockout mouse model and show a sex-specific role for epithelial *Stat3* signaling in *K-ras*-mutant LUAD.

## Introduction

Lung cancer is the leading cause of cancer deaths worldwide^1^. Non-small cell lung cancer (NSCLC) represents the major histological type of lung malignancy diagnosed^2^. Lung adenocarcinoma (LUAD) is the most common histological subtype of NSCLC accounting for over 50% of diagnosed lung cancer cases^2,3^. The Kirsten rat sarcoma viral oncogene (*K-ras*) is the most commonly mutated oncogene in LUADs (~25–30%) particularly in those that developed in lifetime smokers^4,5^. Compared to other solid tumors and molecular lung cancer subtypes, *K-ras* mutant LUAD displays a dismal prognosis and is resistant to most forms of systemic or targeted therapies^5^. These facts warrant the urgent need to develop new or improved strategies for early treatment of *K-ras* mutant LUAD—advances that heavily rest on understanding molecular underpinnings of this particular type of lung malignancy.

Accumulating evidence suggests that tumor-promoting inflammation is a major hallmark of cancer^6,7^. Interestingly, LUAD patients with increased serum levels of the inflammatory cytokine IL-6^8–11^ and high numbers of inflammatory cells in the lung tumor microenvironment (TME) were shown to exhibit a relatively poor prognosis^12,13^. We and others have demonstrated that inflammatory cytokines (e.g. IL-6) can reprogram the lung TME and promote lung tumorigenesis^6,14–16^. In this context, a better understanding of the role of inflammation and the immune microenvironment in lung carcinogenesis may shed light on new high-potential targets for therapy (e.g. immune-based therapy).

Earlier work demonstrated that the proliferative, survival, and angiogenic effects of IL-6 on epithelial cells are mediated by the STAT3 pathway^17,18^. Activation of STAT3, an IL-6-responsive transcription factor, was shown to induce tumor-promoting inflammation as well as activate canonical oncogenic pathways^18,19^. In our previous work, we revealed a crucial role for IL-6-mediated signaling in *K-ras* mutant lung tumorigenesis^16^ using a mouse model we had previously developed in which a mutated form of *K-ras* was expressed specifically in airway cells under the control of the club cell secretory protein (CCSP) promoter^20^. The STAT3 pathway was found to be aberrantly activated during the development of *K-ras* mutant lung tumors in this model, and this activity was attenuated by treatment with an antibody against IL-6^16^, suggesting a crucial role for inflammation through IL-6/STAT3 signaling in *K-ras-*induced tumorigenesis. Yet, STAT3-dependent contextual cues in the pathogenesis of *K-ras* mutant lung malignancy remain largely unexplored.

To better understand the role of the STAT3 pathway in *K-ras* mutant lung tumorigenesis, we here derive a lung epithelial-specific *K-ras* mutant/*Stat3* conditional knockout (LR/*Stat3*^Δ/Δ^) mouse model. We find that deletion of epithelial *Stat3* decreased *K-ras* mutant-driven lung tumorigenesis in female mice, yet led to a surprising outcome in male littermates, who exhibit the opposite effect of enhanced malignancy. Functional pathway and immune TME analyses reveal differential immune phenotypes among *Stat3*-deleted female and male mice carrying the *K-ras* mutation. Furthermore, we demonstrate that inhibition of estrogen signaling in female mice augments *K-ras* mutant lung cancer development. Our data reveal markedly disparate sex contextual effects on *K-ras* mutant lung cancer development via differential reprogramming of lung onco- and immune- phenotypes, thus providing insights into potential new strategies for personalized (e.g. sex-based) immunotherapy.

## Results

### Sex-differential effects of *Stat3* deletion on lung tumor

**Fig. 1:**
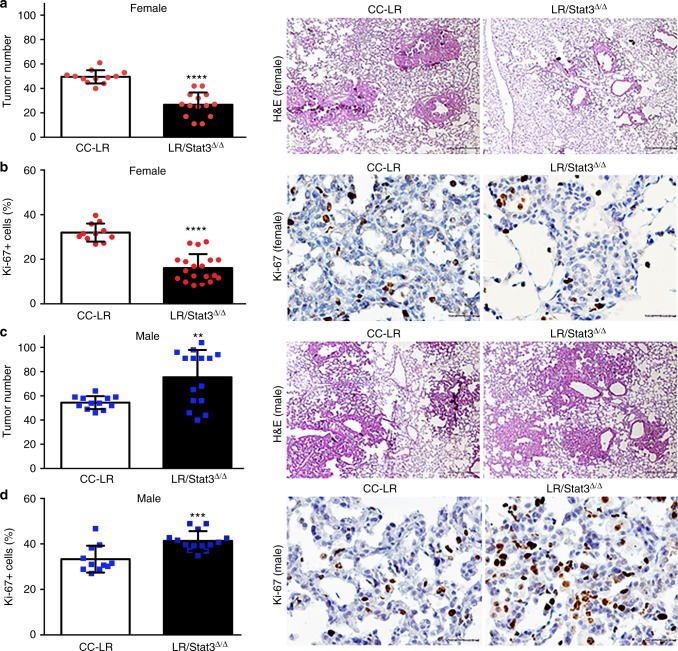
Epithelial *Stat3* deletion induces sex-associated differences in *K-ras* mutant tumor burden. Lung surface tumor number (left panel) and histopathologic appearance (40× magnification, scale bar = 100 μm) of the lung in female (red circles, *n* = 12–14) (**a**) and male (blue squares, *n* = 12–15) (**c**) CC-LR (middle panel) and LR/*Stat3*^Δ/Δ^ (right panel) mice at the age of 14 weeks. Quantitative analysis (left panel) and representative lung photomicrographs (20× magnification, scale bar = 50 μm) of positive tumor cells for Ki-67 in female (*n* = 11–14) (**b**) and male (*n* = 12–14) (**d**) CC-LR (middle panel) and LR/*Stat3*^Δ/Δ^ (right panel) mice at the age of 14 weeks. (Data represent means ± standard error of mean (SEM); ^****^P < 0.0001; ^***^P < 0.001; ^**^P < 0.005 using two-tailed *t*-test, experimental replicate # 3–4)

We previously revealed a crucial role for IL-6-mediated signaling as well as aberrant *Stat3* activity in the pathogenesis of *K-ras* mutant lung cancer^16,20^. Yet, *Stat3*-dependent contextual cues in the pathogenesis of *K-ras* mutant lung malignancy remain largely unknown. To fill this void, we derived CC-LR mice with conditional deletion of *Stat3* in epithelial cells (LR/*Stat3*^Δ/Δ^ mice). We then compared lung tumorigenesis in CC-LR and LR/*Stat3*^Δ/Δ^ mice. Initial analyses revealed no significant differences in tumor number between CC-LR and LR/*Stat3*^Δ/Δ^ mice. Further analysis, however, demonstrated remarkable sex-differential (i.e., disparity) tumor development in LR/*Stat3*^Δ/Δ^ mice relative to CC-LR littermates evidenced by disparate tumor numbers (Fig. 1) and areas (Supplementary Figure 1). We found that while female LR/*Stat3*^Δ/Δ^ mice exhibited significantly lower tumor burdens including proliferating lesions, evidenced by Ki-67 positivity, compared to female CC-LR mice (Fig. 1a, b), the opposite was true of male littermates (Fig. 1c, d). Male mice with deletion of epithelial *Stat3* displayed elevated tumor burdens and Ki-67 immunoreactivity compared to male CC-LR animals (Fig. 1c, d). Additionally, lungs of female LR/*Stat3*^Δ/Δ^ mice exhibited reduced expression of the angiogenic marker, CD31 protein, relative to CC-LR littermates, whereas male LR/*Stat3*^Δ/Δ^ mice exhibited augmented CD31 amounts (Supplementary Figure 2). These findings demonstrate that epithelial *Stat3* functions in a sex-dependent manner in *K-ras* mutant lung tumorigenesis.

### Sex-specific immune programs associated with *Stat3* deletion

**Fig. 2:**
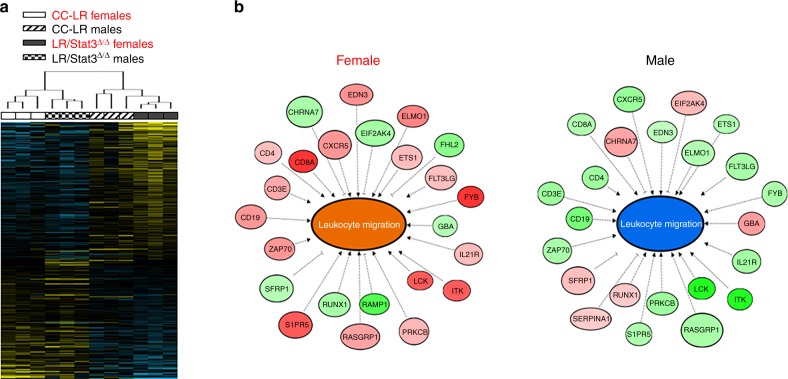
Sex-differential immune expression programs in lungs of epithelial-specific *K-ras* mutant *Stat3* deficient mice. **a** Whole-transcriptome sequencing of whole lungs from 14-week-old CC-LR and LR/*Stat3*^Δ/Δ^ male and female mice (*n* = 3 within each genotype and sex group; *n* = 12 total) was performed using the Ion Torrent Proton platform and as described in the Methods section. Differentially expressed transcripts (*n* = 339) were identified using a mixed-effects model as described in the Methods section and analyzed by clustering. Rows represent gene features and columns denote samples (yellow, upregulated compared to median sampled; blue, downregulated expression). **b** Differentially expressed transcripts were functionally and topologically analyzed by pathways and gene set analysis using IPA as described in the Methods section. The gene–gene interaction networks depict significantly predicted inhibition of leukocyte migration and associated gene sets in *Stat3*-deleted males with the opposite pattern in the female counterparts (orange, activated molecular function; blue, inhibited molecular function; red, upregulated expression; green, downregulated expression)

Our findings on stark sex-associated differences in the effect of epithelial *Stat3* deletion on *K-ras* mutant lung tumorigenesis prompted us to survey global gene expression programs and signaling cues downstream of epithelial *Stat3*. We performed whole-transcriptome sequencing (RNA-Seq) of total lungs from male and female CC-LR and LR/*Stat3*^Δ/Δ^ mice at the age of 14 weeks (*n* = 3 each group; *n* = 12 total). Using the Ion Torrent Proton, we sequenced an average of 42 million reads per specimen (Supplementary Table 1). To determine sex-dependent differentially expressed transcripts between LR/*Stat3*^Δ/Δ^ and CC-LR mice, we employed a mixed effects model with terms for genotype and sex modeling in the interaction between the two factors (see Methods section). Using a *P* *<* 0.01 (mixed effects-model/ANOVA), we identified 339 transcripts that were differentially regulated among females and males in LR/*Stat3*^Δ/Δ^ relative to CC-LR mice (Supplementary Data 1). Hierarchical cluster analysis revealed sex-differential effects of the identified differentially expressed transcripts evidenced by co-clustering of male CC-LR mice with female LR/*Stat3*^Δ/Δ^ counterparts as well as female CC-LR mice with male LR/*Stat3*^Δ/Δ^ animals (Fig. 2a).

We then sought to gain insights into sex-associated functional signaling cues downstream of epithelial *Stat3* deletion. We performed paired pathways and genes set enrichment analyses of the identified 339 transcripts independently in males and females followed by cross-comparison of both functional interrogations. These functional interrogations revealed largely disparate pathway and gene set deregulation following epithelial *Stat3* deletion in female LR/*Stat3*^Δ/Δ^ mice relative to male littermates (Supplementary Tables 2 and 3). Pathways analysis revealed significant predicted activation of signaling pathways mediated by *Nfat* and phospholipase C (all *P* < 0.01, Fisher’s test) following epithelial *Stat3* deletion in female CC-LR mice as opposed to stipulated inhibition of these pathways in male LR/*Stat3*^Δ/Δ^ counterparts (Supplementary Table 2). Gene set enrichment analysis also demonstrated predicted activation of canonical pro-tumor genes or sets following epithelial *Stat3* deletion in males including cyclin D1 (*CCND1*; *z*-score = 1.4), c-AMP response element binding protein 1 (*CREB1*; *z*-score = 2.0), and insulin growth factor receptor 1 (IGFR1; *z*-score = 1.9) (all *P* < 0.05 of the Fisher’s test), with all these genes predicted to be inhibited in female LR/*Stat3*^Δ/Δ^ counterparts (Supplementary Table 3). Gene set enrichment analysis also revealed predicted activation of T-cell receptor (TCR) signaling (*z*-score = 2.6; *P* = 0.003 of the Fisher’s test) following epithelial *Stat3* deletion in female CC-LR mice with the opposite pattern (inhibition of TCR signaling; *z*-core = −2.0) occurring in male LR/*Stat3*^Δ/Δ^ littermates (Supplementary Table 3). Of note, further analysis linking differential gene expression and aberrant biological functions revealed a modulation of various gene sets overall pointing to marked dampening of the host anti-tumor immune response (e.g. gene sets associated with leukocyte migration) following *Stat3* deletion in male CC-LR mice (Fig. 2b, Supplementary Data 2). In stark contrast, gene set analysis revealed increased activation of the anti-tumor immune response in female LR/*Stat3*^Δ/Δ^ mice relative to CC-LR mice of the same sex (Fig. 2b, Supplementary Data 2). Genes typifying attenuated leukocyte migration in male LR/*Stat3*^Δ/Δ^ mice included decreased expression of *Cd4*, *Cd8a*, *Cd19*, and *Cd3e* with opposite patterns of expression (upregulation of those genes) in female counterparts (Fig. 2b). These data point to differential modulation of the host anti-tumor immune response following epithelial *Stat3* deletion and during *K-ras* mutant lung tumorigenesis in female (activated) and male (inhibited) CC-LR mice.

### Interplay between sex, *Stat3*, and lung TME

**Fig. 3:**
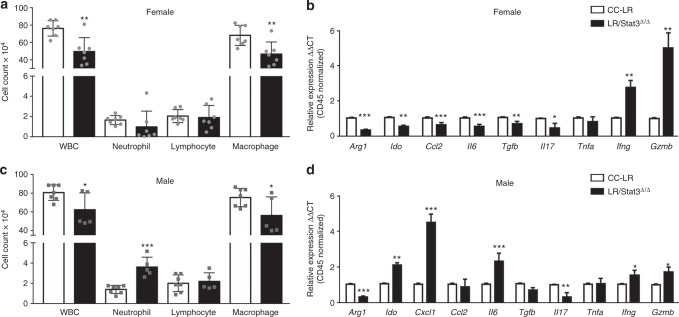
Epithelial *Stat3* deletion changes the lung tumor microenvironment in a sex-dependent manner. Total inflammatory cell and lineage-specific leukocyte numbers from bronchoalveolar lavage fluid (BALF) of female (*n* = 7) (**a**) and male (*n* = 5–7) (**c**) CC-LR and LR/*Stat3*^Δ/Δ^ mice at the age of 14 weeks. Relative expression of *Arg1*, *Ido*, *Cxcl1*, *Ccl2*, *Il6*, *Tgfb*, *Il17*, *Tnfa*, *Ifng* and *Gzmb* mRNA in whole lungs of female (**b**) and male (**d**) CC-LR and LR/*Stat3*^*Δ*/Δ^ mice at the age of 14 weeks, normalized by CD45 expression (data represent means ± SEM; ^***^P < 0.001; ^**^P < 0.005; ^*^P < 0.05 using two-tailed *t*-test, experimental replicate # 2-3). WBC, white blood cell

Our RNA-Seq data prompted us to survey the effects of epithelial *Stat3* deletion on the lung TME, in male and female CC-LR mice. We determined immune cellular composition in bronchoalveolar lavage fluid (BALF) from 14-week-old female tumor-bearing littermates. This analysis showed that the female LR/*Stat3*^Δ/Δ^ mice relative to CC-LR littermates exhibited significantly reduced numbers of white blood cells (WBCs), including macrophages (Fig. 3a). No changes were observed in neutrophil or lymphocyte counts in female LR/*Stat3*^Δ/Δ^ mice versus CC-LR littermates (Fig. 3a). We next measured by quantitative real-time PCR the expression levels of a panel of inflammatory genes in the whole lung normalized to expression of CD45, a pan-hematopoietic marker (Fig. 3b). We found significantly reduced expression of immunosuppressive genes, arginase 1 (*Arg1*) and indoleamine 2,3-dioxygenase (*Ido*), in female LR/*Stat3*^Δ/Δ^ mice relative to CC-LR littermates (Fig. 3b) suggestive of reduced pro-tumor type 2 macrophage polarization and myeloid derived suppressor cells (MDSCs) responses and enhanced T-cell activation. Female LR/*Stat3*^Δ/Δ^ mice also concurrently exhibited significantly decreased expression levels of the macrophage chemoattractant chemokine ligand 2 (*Ccl2*) and of the interleukin 6 (*Il6*) cytokine (Fig. 3b)—in accordance with the observed decrease in the number of macrophages observed (Fig. 3a). Our expression analysis also pointed to attenuated expression of the immunosuppressive cytokine transforming growth factor beta (*Tgfb*) and of *Il17* in female LR/*Stat3*^Δ/Δ^ mice relative to CC-LR littermates which in combination with reduced *Il6* expression are suggestive of decreased T regulatory (Treg) and T helper 17 (Th17) cells infiltration or differentiation. Furthermore, our data revealed increased expression of interferon gamma (*Ifng*) and granzyme B (*Gzmb*) in female LR/*Stat3*^Δ/Δ^ mice (Fig. 3b), suggesting anti-tumor immune responses in *Stat3*-deleted females. Similar to female LR/*Stat3*^Δ/Δ^ mice, BALF of male *Stat3*-deleted counterparts also exhibited significantly reduced numbers of total WBCs and macrophages (Fig. 3c). Interestingly, BALF of male LR/*Stat3*^Δ/Δ^ mice relative to male CC-LR littermates exhibited significantly increased levels of neutrophils (Fig. 3c). This effect was accompanied by increased expression of the *Cxcl1* gene in male LR/*Stat3*^Δ/Δ^ mice (Fig. 3d). Of note and in sharp contrast to female LR/*Stat3*^Δ/Δ^ counterparts, BALF of male LR/*Stat3*^Δ/Δ^ mice exhibited significantly elevated levels of *Ido* and *Il6*, and decreased expression of *Ifng* and *Gzmb* (Fig. 3d). No changes were observed in *Ccl2*, *Tgfb*, and *Tnfa* expression in male LR/*Stat3*^Δ/Δ^ mice (Fig. 3d). These results suggest increased protumor (type 2) immune responses (M2 macrophage polarization, induction of MDSCs and Treg responses) and decreased Th1 and CD8 T-cell activation in male LR/*STAT3*^Δ/Δ^ mice (Fig. 3d) relative to female *Stat3*-deleted counterparts (Fig. 3b). Concordantly, we also noted increased NF-κB activation, evidenced by increased p65 DNA-binding activity, in tumors from male LR/*Stat3*^Δ/Δ^ mice relative to male CC-LR littermates (Supplementary Figure 3a) but not in female LR/*Stat3*^Δ/Δ^ counterparts (Supplementary Figure 3b).

**Fig. 4:**
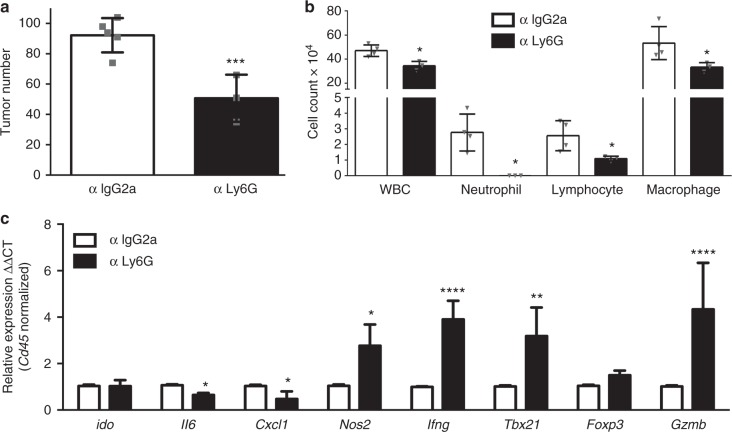
Neutrophil depletion inhibits *K-ras* mutant lung tumors in male LR/*Stat3*^Δ/Δ^ mice. **a** Lung surface tumor number in 14-week-old male LR/*Stat3*^Δ/Δ^ mice treated with control (IgG2) (*n* = 4) or anti-Ly6G (*n* = 4) antibody. **b** Total inflammatory cell and lineage-specific leukocyte numbers from BALF of control (*n* = 4) or anti-Ly6G antibody treated (*n* = 4) male LR/*Stat3*^Δ/Δ^ mice. **c** Relative expression of *Arg1*, *Ido*, *Cxcl1*, *Ccl2*, *Il6*, *Tgfb*, *Il17*, *Tnfa*, *Ifng* and *Gzmb* mRNA in whole lungs, normalized by CD45 expression, of LR/*Stat3*^Δ/Δ^ male mice treated with control (*n* = 4) or anti-Ly6G (*n* = 4) antibody (data represent means ± SEM; ^***^P < 0.001; ^**^P *<* 0.005; ^*^P < 0.05 using two-tailed *t*-test, experimental replicate # 2-3). WBC, white blood cells

Based on our finding on the increased number of infiltrated neutrophils in male LR/*Stat3*^Δ/Δ^ mice, we sought to probe the specific role of these immune cells in *K-ras* mutant lung tumor development following *Stat3* deletion. As expected, treatment of male LR/*Stat3*^Δ/Δ^ mice with anti-Ly6G completely abrogated neutrophils and decreased total WBC counts (Fig. 4b). This effect was accompanied by reduced numbers of lung macrophages and lymphocytes following anti-Ly6G treatment (Fig. 4b). Of note, depletion of neutrophils using an anti-Ly6G antibody in male LR/*Stat3*^Δ/Δ^ mice significantly decreased the tumor burden when compared with isotype control-treated mice (IgG2) (Fig. 4a). This effect was accompanied by significantly decreased expression of *Il6* and *Cxcl1* levels as well as markedly and significantly increased expression of the anti-tumor and/or cytotoxic immune genes nitric oxide synthase 2 (*Nos2*), *Ifng*, T-box transcription factor 21 (*Tbx21*), and *Gzmb* (Fig. 4c). These findings suggest that epithelial *Stat3* deletion reprograms the *K-ras* mutant lung TME in a sex-differential manner via NF-κB-mediated induction of a CXCL1-driven neutrophilic response.

### Anti-tumor effects of IL-6 blockade in male LR/*Stat3*^Δ/Δ^ mice

**Fig. 5:**
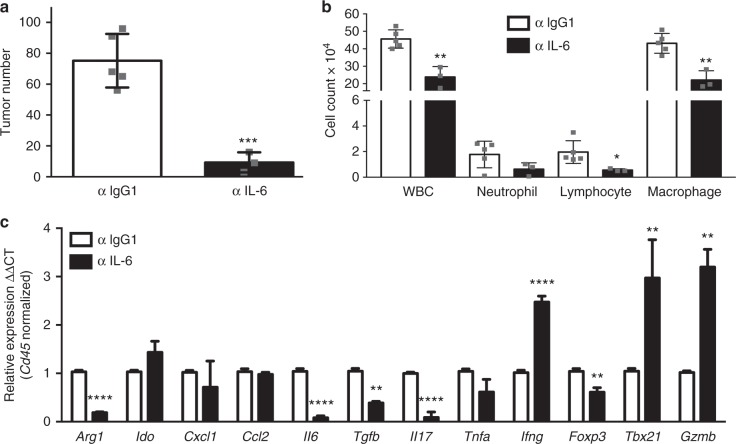
IL-6 blockade reduces *K-ras*-induced lung tumorigenesis in male LR/*Stat3*^Δ/Δ^ mice. **a** Lung surface tumor number in 14-week-old male LR/*Stat3*^Δ/Δ^ mice after treatment with control (IgG1) (*n* = 5) or anti-IL-6 (*n* = 3) antibody. **b** Total inflammatory cell and lineage-specific leukocyte numbers from BALF of control (*n* = 5) or anti-IL-6-treated (*n* = 3) male LR/*Stat3*^Δ/Δ^ mice. **c** Relative expression of *Arg1*, *Ido*, *Cxcl1*, *Ccl2*, *Il6*, *Tgfb*, *Il17*, *Tnfa*, *Ifng*, *Foxp3*, *Tbx21*, and *Gzmb* mRNA in whole lungs, normalized by CD45 expression, of male LR/*Stat3*^Δ/Δ^ mice after treatment with control (*n* = 5) or anti-IL-6 (*n* = 3) antibody (data represent means ± SEM; ^**^P < 0.005, ^*^P < 0.05 using two-tailed *t*-test, experimental replicate # 2–3)

Our data demonstrating increased levels of *Il6* expression in male LR/*Stat3*^Δ/Δ^ mice relative to both female LR/*Stat3*^Δ/Δ^ counterparts and male CC-LR littermates prompted us to assess the effects of IL-6 blockade on lung tumor development and the immune microenvironment—in the context of epithelial *Stat3* deletion and male sex. We treated male LR/*Stat3*^Δ/Δ^ mice with a monoclonal antibody against IL-6 for 8 weeks, prior to lung and BALF collection. We observed that male LR/*Stat3*^Δ/Δ^ mice treated with anti-IL-6 antibody exhibited significantly decreased lung tumor burdens compared with male littermates treated with control IgG1 antibody (Fig. 5a). This effect was associated with a significant reduction of total WBC counts assessed in BALF of male LR/*Stat3*^Δ/Δ^ mice (Fig. 5b). Treatment with anti-IL-6 antibody led to significant decreases in the number of lymphocytes as well as macrophages along with a reduction in the number of neutrophils albeit not reaching statistical significance (Fig. 5b). Gene expression analysis of immune cells in the whole lung demonstrated significantly reduced levels of immune markers indicative of an immune-suppressive protumor (type 2) microenvironment, e.g., *Arg1*, *Tgfb*, *Il17*, *Tnfa,* and *Foxp3*, in anti-IL-6-treated male LR/*Stat3*^Δ/Δ^ mice relative to littermates treated with control IgG1 antibody (Fig. 5c). Conversely, treatment of male LR/*Stat3*^Δ/Δ^ mice with anti-IL-6 antibody resulted in significantly increased expression levels of immune markers indicative of M1 macrophage polarization, Th1 differentiation and/or cytotoxic (CD8 T/NK cell) anti-tumor response (*Ifng*, *Tbx21*, and *Gzmb*) (Fig. 5c). Of note, IL-6 blockade did not alter the expression levels of *Ido*, *Cxcl1*, and *Ccl2*; however, interestingly treatment with anti-IL-6 antibody suppressed *Il6* expression (Fig. 5c). These findings suggest that IL-6 signaling promotes *K-ras* mutant lung tumorigenesis downstream of epithelial *Stat3* deletion in male mice and blockade of the cytokine reformats the lung TME toward an anti-tumor phenotype.

### Effects of estrogen receptor blockade in LR/*Stat3*^Δ/Δ^ females

**Fig. 6:**
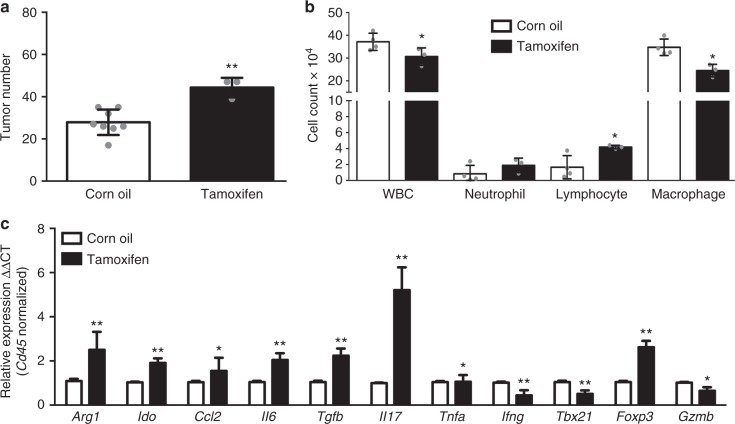
Estrogen receptor blockade promotes lung tumorigenesis in female LR/*Stat3*^Δ/Δ^ mice. **a** Lung surface tumor number in 14-week-old female LR/*Stat3*^Δ/Δ^ mice after corn oil (*n* = 8) or tamoxifen (*n* = 3) treatment. **b** Total inflammatory cell and lineage-specific leukocyte numbers from BALF of female LR/*Stat3*^Δ/Δ^ mice after corn oil (*n* = 4) or tamoxifen (*n* = 3) treatment. **c** Relative expression of *Arg1*, *Ido*, *Ccl2*, *Il6*, *Tgfb*, *Il17*, *Tnfa*, *Ifng*, *Tbx21*, *Foxp3*, and *Gzmb* mRNA in whole lungs, normalized by CD45 expression, of female LR/*Stat3*^Δ/Δ^ mice after corn oil (*n* = 4) or tamoxifen (*n* = 3) treatment (data represent means ± SEM; ^**^P < 0.005, ^*^P < 0.05 using two-tailed *t*-test, experimental replicate # 2-3)

We surmised that aberrant sex hormone signaling may be implicated in the observed sex-associated differences in *K-ras* mutant lung tumorigenesis in the context of epithelial *Stat3* deletion. To probe this supposition, we blocked estrogen receptor (ER) signaling by tamoxifen in female LR/*Stat3*^Δ/Δ^ mice. We observed that tamoxifen-treated female LR/*Stat3*^Δ/Δ^ mice exhibited significantly elevated tumor burdens in comparison to control mice (Fig. 6a). This effect was associated with significantly decreased WBC counts, including macrophages, whereas lymphocyte and neutrophil counts were increased (Fig. 6b). In line with these observations, tamoxifen treatment of female LR/*Stat3*^Δ/Δ^ mice overall resulted in increased levels of immune markers in whole lung indicative of immune suppression and a protumor response (e.g. *Arg1*, *Ccl2*, *Il6*, *Tgfb, Il17, Foxp3, Ido*) in comparison with controls. Tamoxifen treatment also resulted in significantly decreased expression of immune genes implicated in Th1 differentiation and the cytotoxic anti-tumor response such as *Ifng*, *Tbx21*, and *Gzmb* (Fig. 6c). Of note, we further examined our RNA-Seq data, probing for functional topological gene networks that were predicted to be significantly modulated following epithelial *Stat3* deletion in males and females. A mechanistic network comprising beta-estradiol signaling (*P*-value of overlap = 6.8 × 10^−5^; Supplementary Figure 4) was activated in female mice with epithelial *Stat3* deletion and that comprised elevated expression of T-cell markers (CD4 and CD3); suggesting potential cues linking estrogen signaling activation and anti-tumor immune mechanisms. Interestingly, analysis of tyrosine phosphorylated STAT3 (pSTAT3) and estrogen receptor beta (ERβ) by IHC in human LUADs demonstrated a significant positive correlation between pSTAT3 and ERβ in females (*P* = 0.04) but not in males (*P* = 0.17) (Supplementary Figure 5). Overall, these results suggest a critical role for estrogen signaling in maintaining an anti-tumor microenvironment, thereby decreasing *K-ras* lung tumor development following *Stat3* deletion in female mice.

## Discussion

**Fig. 7:**
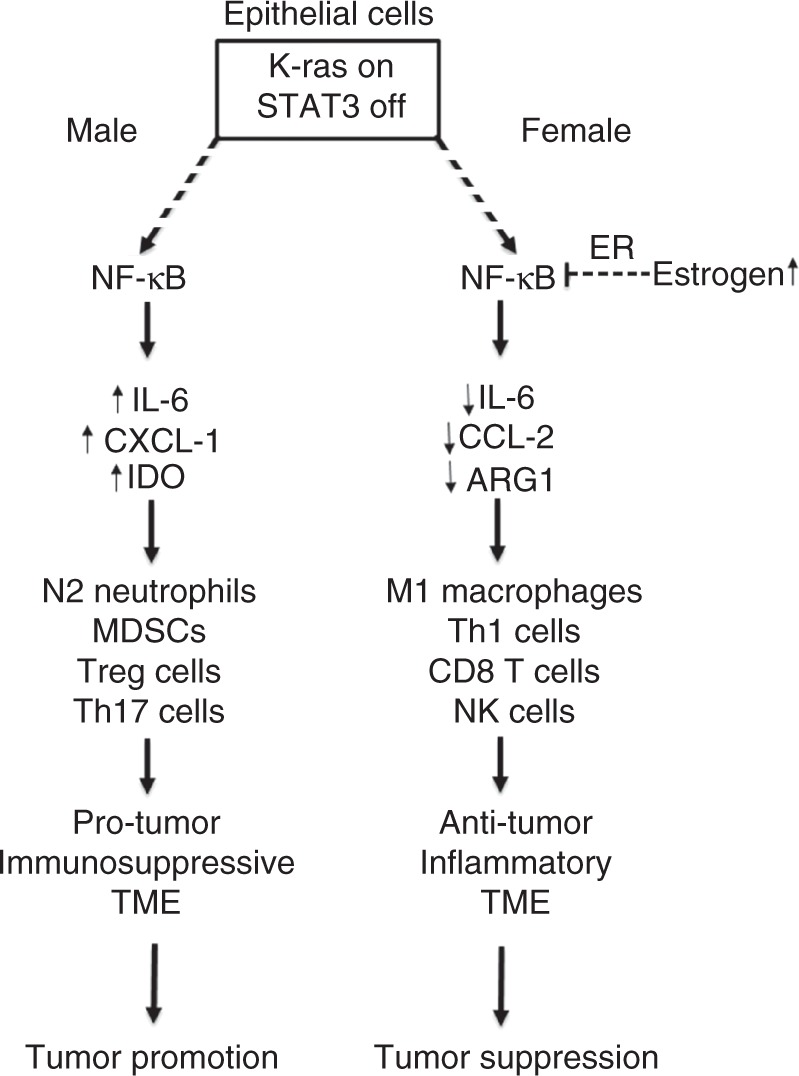
Mechanisms underlying sex-associated function of epithelial STAT3 signaling in *K-ras* mutant lung cancer. Our findings suggest that there is a differential regulation of NF-κB activation in K-ras mutant STAT3-deficient lung epithelium within sex, and estrogen signaling plays a protective role in females by reducing the activation of NF-κB pathway and subsequent induction of an anti-tumor immune response. It also suggests that activation of NF-κB pathway in the lung of male mice drives the tumor-promoting effect of STAT3 deficiency probably by induction of IL-6 and CXCL1, and subsequent recruitment of neutrophils and induction of a pro-tumor immunosuppressive response

Here we sought to understand the role of the epithelial STAT3 pathway in the development of *K-ras* mutant lung cancer. Toward this, we developed and studied a *K-ras* mutant induced mouse model (CC-LR) with conditional deletion of epithelial specific *Stat3* (LR/*Stat3*^Δ/Δ^). Interestingly, we discovered marked sex-associated differences in lung tumor burdens due to the lack of epithelial *Stat3*—tumor burden was significantly reduced in female LR/*Stat3*^Δ/Δ^ mice whereas tumorigenesis was markedly augmented in male counterparts. Whole-transcriptome analysis by RNA-Seq revealed a notable differential modulation of the host immune response among female and male LR/*Stat3*^Δ/Δ^ mice. The sex-associated differences in tumor burden were concordantly accompanied by enhanced anti-tumor immune responses in female LR/*Stat3*^Δ/Δ^ mice and, conversely, elevated pro-tumor immune phenotypes in the male counterparts. Additional immune phenotype analysis suggested that augmented pathogenesis of *K-ras* mutant lung tumors in male LR/*Stat3*^Δ/Δ^ mice was, in part, mediated by various immune mechanisms, that are possibly non-mutually exclusive, including increased NF-κB activation, IL-6 induction, and a CXCL1-mediated neutrophilic response. Of note, targeting studies using tamoxifen revealed a crucial role for ER signaling in counteracting *K-ras* mutant lung cancer pathogenesis following *Stat3* deletion in female mice. All in all, our findings demonstrate a critical sex-differential role for epithelial STAT3 signaling in the pathogenesis of *K-ras* mutant lung cancer. We surmise that there exist signaling cues that link activated estrogen signaling and anti-tumor immune responses in the pathogenesis of *K-ras* mutant lung cancer (Fig. 7). Our findings suggest the plausible use of sex-driven personalized immunotherapeutic approaches for this aggressive malignancy.

A major observation of our study was the marked sex disparity in lung oncogenesis following epithelial *Stat3* deletion in mice with induced mutant *K-ras*. This is in line, albeit indirectly, with various earlier human studies demonstrating that male lung cancer patients exhibit relatively poorer prognosis and response to treatment compared to female patients^21,22^. Our observations prompted us to further investigate global cues underlying these disparate sex-associated effects by RNA-Seq. RNA-Seq profiling coupled with functional pathways and gene set enrichment analyses demonstrated differentially expressed profiles, gene sets, and networks that indicated a disparate modulation of the host immune response among male and female LR/*Stat3*^Δ/Δ^ mice. Among these immune gene profiles, we noted that *Il21r*, *Cd19*, *Cd4*, *Cd8*, and *Cd3* were largely augmented in females following *Stat3* deletion, whereas those genes were conversely suppressed in *Stat3*-deleted males. Further analysis by qRT-PCR also showed elevated expression of genes indicative of an anti-tumor immune response such as *Ifng* and *Gzmb* in female LR/*Stat3*^Δ/Δ^ mice. It is noteworthy that earlier studies have shown that human lung tumors with CD4^+^, CD8α^+^, CD3^+^ (T-cell), and B-cell tumor-infiltrating lymphocytes (TILs) overall exhibit better prognosis compared to immunologically mute (cold) tumors^23–26^. Our findings here, in the context of previous reports, strongly suggest that the lungs of female LR/*Stat3*^Δ/Δ^ mice exhibit an anti-tumor host immune response.

In sharp contrast to their female counterparts, we found that male LR/*Stat3*^Δ/Δ^ mice exhibited largely increased expression of *Il6* compared to male CC-LR mice. Further, blockade of IL-6 in male mice significantly inhibited lung tumorigenesis with concomitant reformatting of the lung TME toward an anti-tumor phenotype characterized by an increased expression of the cytotoxic immune response (e.g. *Gzmb*) and a decreased expression of *Tgfb* and *Foxp3*, markers of tumor-promoting Treg response^27^. It is important to mention that in the mouse models we employed, *Stat3* was conditionally and selectively depleted only in airway epithelium. Thus, it is plausible and likely that the tumor stroma and immune microenvironment still contains *Stat3* which may be functionally targeted by IL-6 blockade. Indeed previous studies^10^, as well as our prior^16^ and present findings, point to crucial tumor-promoting roles for IL-6/STAT3 signaling, including that in the TME, in the pathogenesis of *K-ras* mutant lung malignancy. A parallel observation from our immune profiling analysis was that the lungs of male LR/*Stat3*^Δ/Δ^ mice exhibited increased expression levels of neutrophil chemoattractant, *Cxcl1*, in comparison to male CC-LR mice. Also, depletion of neutrophils by anti-Ly6G antibody significantly reduced lung tumorigenesis while reformatting the lung TME toward an anti-tumor phenotype. These findings are in line with previous studies showing the role of neutrophils, the dominant suppressive immune cell population in human NSCLC^28^, in supporting cancer development and progression in murine *K-ras* mutant lung cancer^29–32^. It is important to note that NF-κB activation promotes carcinogenesis largely by transactivation of a battery of chemokines and cytokines including IL-6, CXCL1, and CCL2^33–36^. Indeed, further analysis in our study revealed that male, but not female, *K-ras* mutant induced mice with *Stat3* deletion exhibited elevated p65 transcriptional activity, indicative of NF-κB activation. Our findings are in line with previous reports demonstrating that NF-κB signaling is activated in *K-ras* mutant lung cancer^36,37^. It is plausible to surmise that NF-κB activity downstream of *K-ras* mutations and *Stat3* deletion is disparate between males (increased activation) and females (decreased NF-κB activity) and this may be in part due to differential activation of estrogen signaling (Fig. 7). Our findings strongly suggest that the TME in female LR/*Stat3*^Δ/Δ^ mice is primed toward an anti-tumor phenotype when compared to female CC-LR mice, whereas the TME in male counterparts is reformatted toward a pro-tumor phenotype.

The role of downstream STAT3 signaling in *K-ras* mutant lung cancer remains poorly understood^18^. Earlier studies have shown that STAT3 mediates tumor-promoting inflammation in part by inhibiting expression of activators of anti-tumor immunity^19,38–40^. On the other hand, the report by Zhou and colleagues demonstrated that the role of STAT3 signaling in lung cancer pathogenesis is contextually dependent on the stage of tumorigenesis with STAT3 exhibiting contrasting roles in tumor initiation and progression^41^. Also, inhibition (by genetic targeting) of STAT3 signaling has been shown to promote *K-ras* mutant-induced LUAD initiation and progression in vivo concomitant with elevated IL-8 levels largely due to NF-κB activation; suggesting that STAT3 can exhibit lung tumor-suppressive properties^42^. It is worthwhile to mention that the report by Grabner and colleagues utilized male mice only^42^. Thus, these earlier reported findings on the link between STAT3 signaling, NF-κB activation, and *K-ras* mutant lung cancer pathogenesis are in close agreement with our present data showing increased lung tumor burdens in *K-ras* mutant-induced male mice specifically following epithelial *Stat3* deletion. Our present study further sheds light on the role of STAT3 signaling in *K-ras* mutant lung cancer pathogenesis by highlighting stark sex disparity in this process and showing, in contrast, attenuated tumorigenesis in female mice with *Stat3* deletion. It is important to note that the report by Crncec and colleagues demonstrated sex-specific tumor suppressor and anti-tumor immune roles for another Stat, *Stat1*, in colitis-associated colorectal cancer in male but not female mice^43^. Our findings suggest that, similar to *Stat1*^43^, epithelial *Stat3* may exhibit sex-specific lung tumor suppressor roles in males.

Treatment of female LR/*Stat3*^Δ/Δ^ mice with tamoxifen significantly increased lung tumor burden. These findings underscore an important role for ER signaling in restricting *K-ras* mutant lung tumorigenesis following *Stat3* deletion. Our results are in line, albeit indirectly, with previous studies revealing a positive correlation between tumoral expression of ERβ and relatively favorable overall and disease-free survival^44,45^. Also, ERβ, particularly the subunit 1, was shown to induce apoptosis of NSCLC cells^46^. It is important to mention that despite the increased risk of lung cancer development in peri/post-menopausal women compared to pre-menopausal females^47^, the suggested relationship between sex hormones and lung tumorigenesis remains controversial. Earlier studies have shown that postmenopausal women receiving hormonal replacement therapy (HRT) exhibited reduced risk for lung cancer^48^. On the other hand, other reports demonstrated an increased risk for lung cancer in post-menopausal women receiving HRT as well as relatively poor prognosis among female lung cancer patients treated with hormone replacement^49–51^. Also, earlier studies have suggested a pro-tumorigenic role for estrogen signaling in the pathogenesis of lung cancer^52^. It is worthwhile to mention that there are conflicting reports on the association of ERβ expression in female NSCLC patients with clinical outcome^44,45,53–55^. Estrogen and its receptor ERβ were shown to promote lung tumorigenesis in vivo^56^. Also, cigarette smoke exposure was demonstrated to increase the levels of estrogen metabolites, which in turn were shown to transactivate various oncogenes including *K-ras*^57^. It is noteworthy that previous studies demonstrated differential levels of carcinogenic estrogen metabolites between tumor and normal lung tissue and between males and females^58^. It is possible that these estrogen metabolites are disparately present in lungs of *Stat3*-deleted male and female mice and may underlie the effects of tamoxifen we observed; suppositions that can be further scrutinized in future studies. Reasons underlying the opposing reported observations on the role of estrogen signaling in lung tumorigenesis are not clear. It is plausible to assume that estrogen signaling in the pathogenesis of *K-ras* mutant LUAD particularly may be distinct from that in other subtypes of lung cancer. This supposition can be scrutinized and discrepancies could be resolved by future mechanistic studies. It is also plausible that effects of estrogen signaling on lung tumor pathogenesis need to be evaluated in the context of specific cell signaling pathways or oncophenotypes (e.g. IL-6-STAT3 signaling). Along these lines, we found a significant positive correlation between the protein levels of pSTAT3 and ERβ in female but not male LUADs, suggesting a link between estrogen and STAT3 signaling in females. It is important to note that our study demonstrated that estrogen signaling impinges on the immune TME and, subsequently, on *K-ras* mutant lung tumorigenesis following *Stat3* deletion. Specifically, inhibition of estrogen signaling was accompanied by reformatting the TME toward a pro-tumor phenotype evidenced by upregulated expression of *Il6*, *Tgfb*, and *Il17* among other pro-tumor immune markers. Our findings are in agreement, albeit indirectly, with previous reports that underscored the effects of estrogen in reducing Th17 cell activity^59^ and the production of pro-inflammatory cytokines, including IL-6^60^, as well as increasing IFNγ production, Th1, and CD8 T-cell responses^59^. It is noteworthy, and as mentioned above, we found increased NF-κB activation downstream of *Stat3* deletion in males compared to females. It is conceivable that estrogen signaling may negatively impinge on NF-κB activity, thus, leading to the observed attenuated pro-inflammatory (e.g. IL-6) cues in the lung immune TME in females following *Stat3* deletion (Fig. 7). All in all, dissecting the mechanisms underlying the dynamic interplay between estrogen/ER signaling, KRAS/STAT3 pathways in the lung tumor TME, which are poorly understood, may provide viable targets and strategies for personalizing targeted and immunotherapeutic modalities against *K-ras* mutant lung cancer (e.g. by sex). For example, we can potentially suggest that females with lung tumors comprising *K-ras* mutations and loss of *Stat3* in a pre-menopause stage are not ideal candidates for anti-IL-6 therapy, while it might be a good strategy for males with similar genetic and expression profile. However, when females reach menopause, IL-6 blockade might be an alternative approach to HRT which should be taken with precaution due to its side effects.

Taken together, our study underscores a sex-differential role for epithelial STAT3 signaling in the development of *K-ras* mutant lung cancer. Our findings also highlight that these sex-differential effects were accompanied by disparate host immune responses, characterized by an elevated pro-tumor immune response in males but increased anti-tumor immunity in female LR/*Stat3*^Δ/Δ^ mice. Mechanistically, we found that absence of epithelial STAT3 in males promotes lung tumorigenesis via enhanced IL-6 signaling and neutrophilic inflammation, which in turn is curtailed by estrogen/ER signaling in females. Overall, our study points to novel signaling cues in the interplay between STAT3/NF-κB pathways, estrogen signaling, and the lung tumor immune microenvironment in the pathogenesis of *K-ras* mutant lung cancer, thus offering opportune targets and/or strategies for new personalized therapies against this malignancy using already available agents (e.g. anti-IL-6 antibodies, STAT3 inhibitors, or HRT).

## Methods

### Animal housing and experiments

CCSP^Cre^/LSL-K-ras^G12D^ mice (CC-LR) were generated as previously described^20^. Briefly, this is a mouse generated by crossing a mouse harboring the LSL-K-ras^G12D^ allele with a mouse containing Cre recombinase inserted into the CCSP locus. LR/*Stat3*^Δ/Δ^ mice were generated by crossing Stat3 conditional knockout mice^61^, kindly provided by Dr. John DiGiovanni (the University of Texas at Austin, Austin, TX), with CC-LR mice. All mice were housed in specific pathogen-free conditions and handled in accordance with the institutional animal care and use committee of The University of Texas MD Anderson Cancer Center. Mice were monitored daily for evidence of disease or death.

### Human LUAD analysis

Informed consents were obtained from all the subjects under approved institutional review board protocols at The University of Texas MD Anderson Cancer Center and at the University of Pittsburgh. Messenger RNA of target molecules in association with clinical outcome was studied in available data from the Profiling of Resistance patterns and Oncogenic Signaling Pathways in Evaluation of Cancers of the Thorax (PROSPECT) expression dataset^62^. Tissue microarrays (TMAs) of 118 LUADs (65 female; 53 male) obtained from the University of Pittsburgh were stained for pSTAT3 and ERβ by immunohistochemistry. Using specific antibodies for pSTAT3 (Clone D3A7; 1:400 dilution; Cell Signaling Technology) and ERβ (MCA1974ST; 1:20 dilution; Abd Serotec), pSTAT3 was scored for the percentage of positive tumor cells and the intensity on a scale of 0–3. The H-score of 0–300 was calculated by multiplying the percentage score and the intensity score. ERβ was scored as the total score of both nuclear and cytoplasmic as described previously^54^. Tumors were considered positive for pSTAT3 and ERβ if at least two cores per case were positive. Correlation between pSTAT3 and ERβ was statistically evaluated using Spearman correlation.

### IL-6 blockade

Six-week-old male CC-LR and LR/*Stat3*^Δ/Δ^ mice were injected intraperitoneally (IP) with 20 mg/kg dose of an anti-IL-6 monoclonal (MP520F3, R&D, Minneapolis, MN) or IgG1 Isotype control (Clone: 43414, R&D, Minneapolis, MN) antibodies twice a week for 8 weeks^16^.

### Neutrophil depletion

Six-week-old male CC-LR and LR/Stat3^Δ/Δ^ mice were injected IP with 20 mg/kg dose of anti-mouse Ly6G (Clone: 1A8, catalog no.: BP0075-1, Bioxcell, West Lebanon, NH) antibody or IgG2A (Clone: C1.18.4, catalog no.: BE0085, Bioxcell, West Lebanon, NH) twice a week for 8 weeks.

### ER blockade

Six-week-old female CC-LR and LR/Stat3^Δ/Δ^ mice were injected IP with 10 mg/mL of tamoxifen (Sigma, catalog no.: T5648) diluted in corn oil, daily for 8 weeks.

### Assessment of lung tumor burden and inflammation

Mice were anesthetized by IP injection of Avertin (Sigma), and their tracheas were cannulated with a blind needle of the appropriate size and sutured into place. Lung surface tumor numbers were counted if visible; then in some of the mice, the lungs were perfused with PBS through the right heart, inflated with 10% buffered formalin (Sigma), removed and processed for histological analysis as we had done previously^16^. Multiple hematoxylin and eosin (H&E)-stained slides from each mouse (three mice per group) were scanned and ImageScope 12.3.3 (Leica, Nussloch, Germany) was used to measure areas of tumor and whole lung. Tumor area (*T*) was measured by circling individual tumor spots and lung area (*L*) was determined by circling the whole lung area while major airways, thymus, and heart were excluded. Tumor/lung area percentages were calculated using the respective formula; Tumor/Lung Area =$\frac{{\mathop {\sum}\nolimits_{i = 1}^{i = n} {T_i} }}{L}$ × 100%. In some mice, BALF was obtained by sequentially instilling and collecting two aliquots of 1 mL PBS through a tracheostomy cannula. The lungs were snap frozen and stored for RNA analysis. Total leukocyte count in BALFs was determined using a hemocytometer; differential cell populations were determined by cytocentrifugation of BALF followed by Wright–Giemsa (Sigma, St. Louis, MO) staining.

### Histochemistry/immunostaining

H&E staining was done as we had described previously^16^. The H&E-stained slides were examined by a pathologist blinded to genotype and treatment, and the proliferative lesions of the lungs were evaluated in accordance with the recommendations of the Mouse Models of Human Cancer Consortium^63^. Immunohistochemical (IHC) staining for evaluating the expression of target proteins was performed using Ki-67 (1:200; ab16667; Abcam, MA, USA), and CD31 (1:50, 550274, BD Biosciences, CA, USA) antibodies as done previously^16^. The numbers of labeled positive cells for these markers were quantitated as a fraction of total tumor nuclei per high power field (40×) in 10 fields from three mice of each group. Results were expressed as a percentage of positive cells ± standard error of mean (SEM).

### Quantitative RT-PCR analysis

Total RNA was isolated from the whole lung according to the TRIzol reagent protocol (Invitrogen, NY, USA) and purified by E.Z.N.A. total RNA kit I (OMEGA, GA, USA). Reverse transcription PCR was performed using the qScript cDNA SuperMix (Quanta Biosciences, Gaithersburg, MD). qPCR was performed according to a standard protocol using SYBR Green FastMix, Low ROX (Quanta Biosciences, Gaithersburg, MD) and products measured on an ABI Viia 7 PCR system (ABI, Foster City, CA). Data are presented as fold change between the test groups and controls, as indicated in the figure legends. Gene-specific primers are listed in Supplementary Table 4.

### Measurement of NF-κB activity in lung tissues

Lung tissues were homogenized, and nuclear proteins were isolated according to NE-PER™ Nuclear and Cytoplasmic Extraction Reagent (Thermo Scientific, USA). Nuclear protein concentration was determined using BCA assay (Thermo Scientific, USA). NF-κB (p65) DNA binding activity was determined by NF-κB (p65) Transcription Factor Assay Kit (Cayman Chem, Ann Arbor, MI) according to the manufacturer’s instructions using 20 μg of nuclear extract in duplicates. After overnight incubation, the optical density (OD) for each well was read with a microplate reader set to 450 nm immediately after adding the stop solution, then average OD for each group was plotted.

### RNA-sequencing

Total RNA was purified from mouse tissues using the miRNeasy kit (Qiagen) according to the manufacturer’s instructions. RNA concentrations were determined using the NanoDrop 2000 spectrophotometer (Thermo Fisher) according to the manufacturer’s protocol. RNA quality was assessed by computing RNA integrity numbers (RINs) on the Agilent 2100 Bioanalyzer according to the manufacturer’s instructions. Total RNA (approximately 800 ng) was ribosomal RNA (rRNA) depleted using the Low Input RiboMinus™ Eukaryote System v2 (Thermofisher) and whole-transcriptome libraries were generated using the Ion Total RNA-Seq Kit v2 (Thermofisher) according to the manufacturer’s instructions. Quality and concentrations of libraries were measured using the Agilent DNA 1000 assay and the 2100 Bioanalyzer. Template reactions were prepared on the Ion Chef Instrument using the Ion PI Hi-Q Chef kit (Thermofisher) and loaded onto Ion PI Chips v3 (Thermofisher) for sequencing on an Ion Proton sequencer according to the manufacturer’s protocol. On an average, approximately 42 million reads were sequenced per sample.

### RNA-Seq and differential gene expression analyses

Raw data analysis was performed as described previously^64^ by alignment (mm10) of reads using both the STAR and Bowtie2 2.1.0 algorithms, combining aligned reads from both, and quantification of transcripts using a modified version of the expectation–maximization (E/M) algorithm. Resultant reads per kilobase per million (RPKM) values were first processed by adding a pseudocount to all values with RPKM <1.0 followed by log (base 2) transformation and quantile normalization. To identify transcripts significantly modulated following *Stat3* knockout and differently expressed among female and male mice, a mixed-effects model with terms for *Stat3* genotype (LR/*Stat3*^Δ/Δ^ versus CC-LR) and sex (female versus male) as well as interaction between the two factors (*P* < 0.01; *n* = 339 transcripts, Supplementary Data 1). All analyses and clustering were performed in the R language environment. Functional pathways analysis, including gene set enrichment and gene-gene network analysis, of differentially expressed transcripts was performed using Ingenuity Pathways Analysis.

### Statistical analysis

Data are presented as mean ± SEM. The statistical significance between two groups was calculated by a two-tailed *t*-test or analysis of variance with adjustment for multiple comparisons as applied. Graphpad Prism 7 software was used for each type of analysis. Differences were considered significant for *P* < 0.05.

## Electronic supplementary material

Supplementary Information

Supplementary Data 1

Supplementary Data 2

Description of Additional Supplementary Files

Peer Review File

## Data Availability

Raw and normalized RNA-Seq data were deposited in the gene expression omnibus (GEO) under series GSE109000 (samples GSM2927851–GSM2927862). The authors also declare that all the data supporting the findings of this study are available within the paper and its supplementary information files.
