# Supplementary material for: Sex specific function of epithelial STAT3 signaling in pathogenesis of K-ras mutant lung cancer
Source: Nat Commun. 2018 Nov 2;9:4589. doi: 10.1038/s41467-018-07042-y (PMC6214980; doi:10.1038/s41467-018-07042-y)
Supplement: Supplementary file 1 — Supplementary Information [file 41467_2018_7042_MOESM1_ESM.pdf]

Sex Specific Function of Epithelial STAT3 Signaling in Pathogenesis of *K-ras* Mutant Lung Cancer

Caetano MS and et al.

SUPPLEMENTARY INFORMATION

SUPPLEMENTARY FIGURES

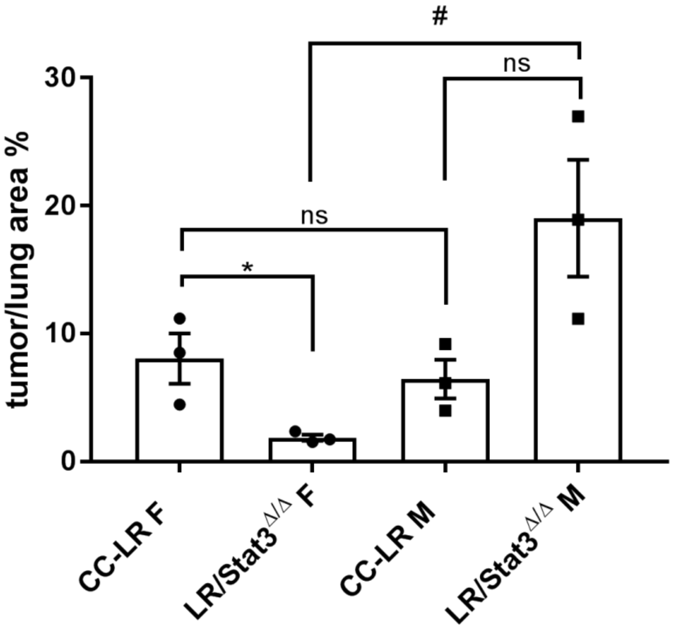

Supplementary Figure 1. Epithelial *Stat3* deletion induces sex-associated discrepancies in the percentage of lung occupied by the tumor. Tumor area in the lungs of female (n = 3) and male (n =3) CC-LR and LR/*Stat3*$^{\Delta/\Delta}$ mice at the age of 14 weeks (Data represent means ± standard error of mean (SEM); ns, non-significant; $^{\#}$ P <0.05; * P < 0.05, using two-tailed t-test).

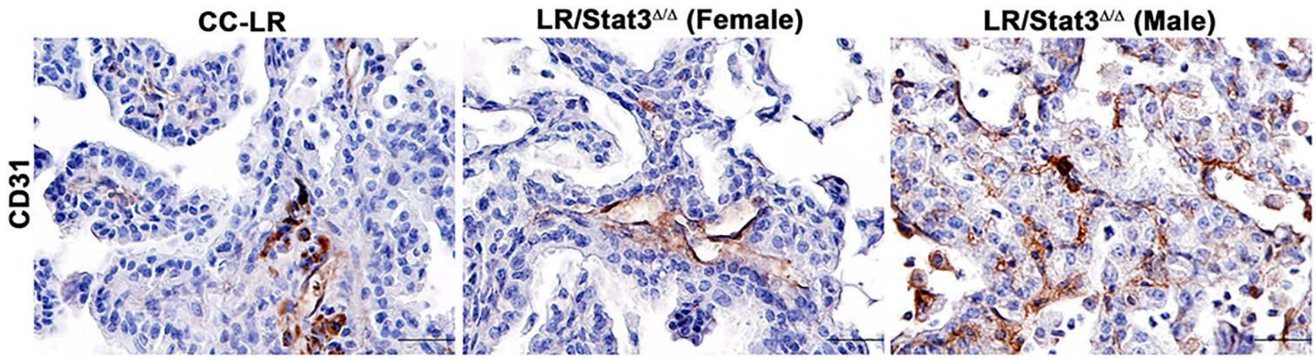

Supplementary Figure 2. Epithelial *Stat3* deletion induces sex-associated discrepancies in CD31 expression. Representative photomicrographs of lung tumors stained for CD31 in CC-LR (left panel) and LR/*Stat3*$^{\Delta/\Delta}$ female (middle panel) and male (right panel) mice at the age of 14 weeks (n = 3) (20X magnification; Scale bar 100μm).

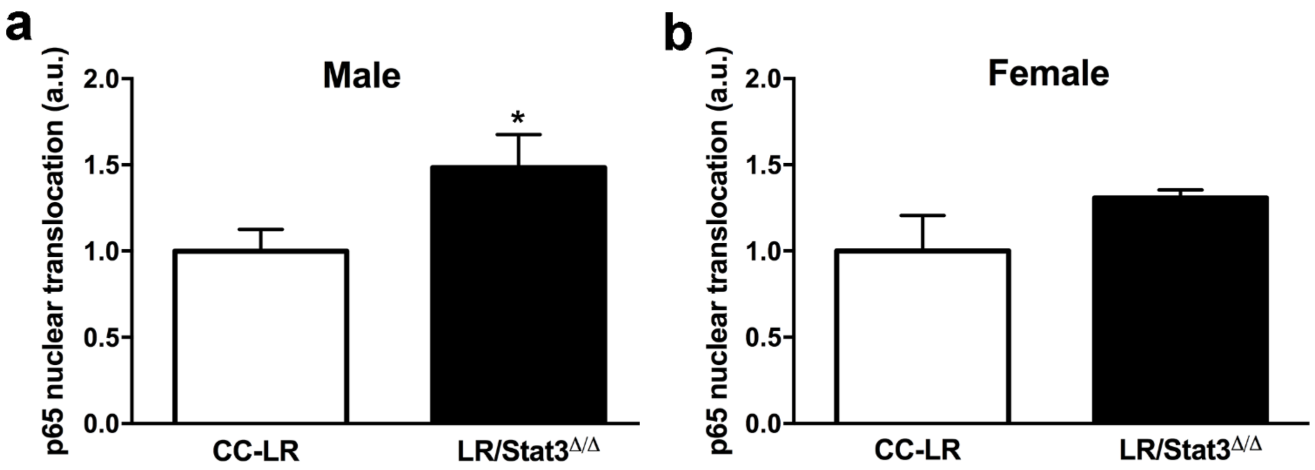

Supplementary Figure 3. NF-κB activation is increased in male, but not female, LR/*Stat3*$^{\Delta/\Delta}$ mice.

NF-κB (P65) DNA binding activity in 14 weeks old male (n = 4) (**a**) and female (n = 4) (**b**) CC-LR and LR/*Stat3*$^{\Delta/\Delta}$ mice ($^{*}P < 0.05$, using two-tailed t-test, experimental replicate # 2).

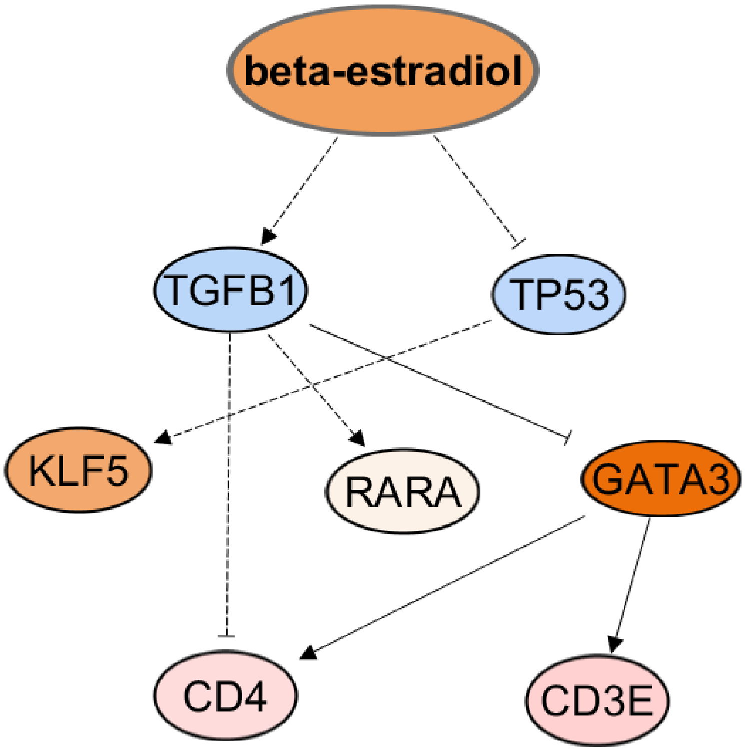

Supplementary Figure 4. Predicted activation of beta-estradiol signaling in female LR/*Stat3*$^{\Delta/\Delta}$ mice compared to their CC-LR female counterparts. Differentially expressed transcripts were topologically organized as functional gene-gene interaction networks using IPA as described in the Methods section. The gene-gene interaction networks depict significantly predicted activation of beta-estradiol signaling (indicated by the orange color) and associated gene nodes in *Stat3*-deleted females compared with their female CC-LR counterparts (orange, predicted activated molecular function; blue, predicted inhibited molecular function; red, up-regulated expression in LR/*Stat3*$^{\Delta/\Delta}$ female mice).

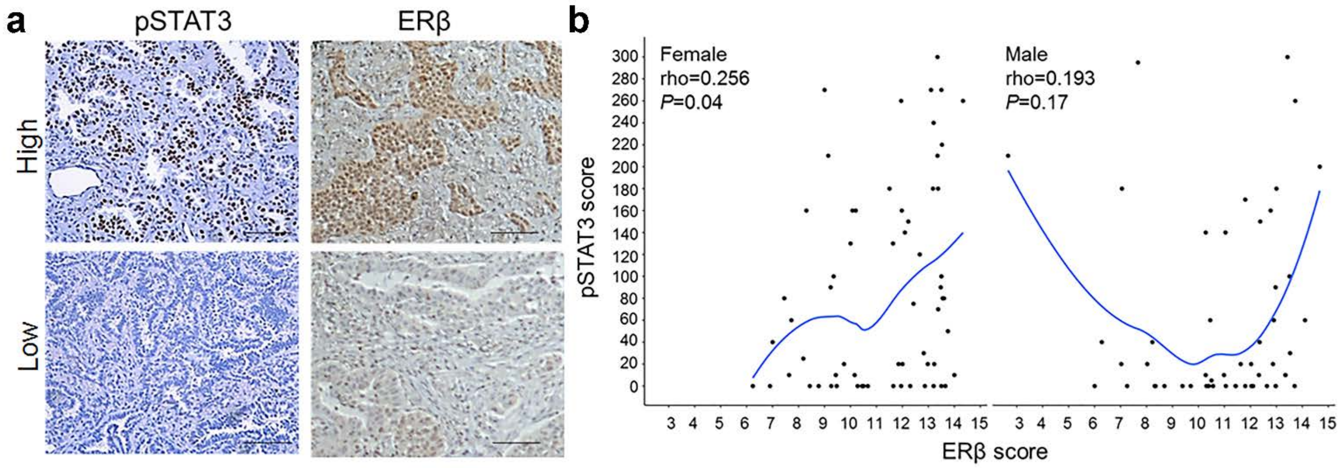

Supplementary Figure 5. Phosphorylated STAT3 levels are significantly correlated with ERβ protein expression in female lung adenocarcinomas. a. Tissue microarrays comprised of formalin-fixed paraffin embedded specimens of 118 LUADs (65 females and 53 males) were evaluated immunohistochemically for protein expression of phosphorylated forms of STAT3 (pSTAT3) and ERβ as described in the Methods section (10X; Scale bar 100μm). Percentage of tumor cells with positive pSTAT3 and ERβ expression along with the intensity of expression was quantified as described in the Methods section. **b**. Correlation between pSTAT3 and ERβ expression was statistically evaluated using Spearman correlation; blue color indicates best fit lines.

SUPPLEMENTARY TABLES

Supplementary Table 1. Number of reads sequenced

Mouse	Genotype	Gender	Reads
CCLR_F1	CC-LR	Female	42091688
CCLR_F2	CC-LR	Female	31998551
CCLR_F3	CC-LR	Female	37142304
CCLR_M1	CC-LR	Male	36088914
CCLR_M2	CC-LR	Male	39675884
CCLR_M3	CC-LR	Male	38198996
LRDD_F1	LR/*Stat3*$^{\Delta/\Delta}$	Female	53125063
LRDD_F2	LR/*Stat3*$^{\Delta/\Delta}$	Female	46495181
LRDD_F3	LR/*Stat3*$^{\Delta/\Delta}$	Female	35335199
LRDD_M1	LR/*Stat3*$^{\Delta/\Delta}$	Male	42559599
LRDD_M2	LR/*Stat3*$^{\Delta/\Delta}$	Male	45994853
LRDD_M3	LR/*Stat3*$^{\Delta/\Delta}$	Male	49724730

CC-LR: *K-ras* mutant-induced mice; LR/Stat3$^{\Delta/\Delta}$: CC-LR mice with epithelial *Stat3* deletion

Supplementary Table 2. Pathways modulated differently following epithelial *Stat3* deletion in lungs of *Kras* mutant-induced female and male mice.

**Canonical pathways**	***P*-value**
T Cell Receptor Signaling	0.000004
Primary Immunodeficiency Signaling	0.000006
Calcium-induced T Lymphocyte Apoptosis	0.000039
Phospholipase C Signaling	0.000046
Role of NFAT in Regulation of the Immune Response	0.000078
CD28 Signaling in T Helper Cells	0.000112
iCOS-iCOSL Signaling in T Helper Cells	0.000372
Acute Myeloid Leukemia Signaling	0.002089
Gαq Signaling	0.002399
PI3K Signaling in B Lymphocytes	0.002692
CCR5 Signaling in Macrophages	0.003311
Maturity Onset Diabetes of Young (MODY) Signaling	0.003311
GM-CSF Signaling	0.004169
Antiproliferative Role of Somatostatin Receptor 2	0.004677
Telomerase Signaling	0.005623
Nitric Oxide Signaling in the Cardiovascular System	0.006166
HGF Signaling	0.006607
NF-κB Activation by Viruses	0.008318
α-Adrenergic Signaling	0.008710
Natural Killer Cell Signaling	0.008913
Breast Cancer Regulation by Stathmin1	0.009772
CXCR4 Signaling	0.010471
Aldosterone Signaling in Epithelial Cells	0.010715
PKCθ Signaling in T Lymphocytes	0.011749
B Cell Development	0.012023
Tec Kinase Signaling	0.012303
p70S6K Signaling	0.012303
Endometrial Cancer Signaling	0.013804
L-cysteine Degradation II	0.014454
CTLA4 Signaling in Cytotoxic T Lymphocytes	0.014791
B Cell Receptor Signaling	0.018621
Thyroid Cancer Signaling	0.019953
Glioma Signaling	0.022387
NRF2-mediated Oxidative Stress Response	0.022909
Neuropathic Pain Signaling In Dorsal Horn Neurons	0.025119
ERK/MAPK Signaling	0.026303
mTOR Signaling	0.026303
Valine Degradation I	0.027542

Protein Kinase A Signaling	0.027542
Regulation of IL-2 Expression in Activated and Anergic T Lymphocytes	0.028184
Cysteine Biosynthesis/Homocysteine Degradation	0.028840
GDP-L-fucose Biosynthesis I (from GDP-D-mannose)	0.028840
Thrombin Signaling	0.028840
GADD45 Signaling	0.030200
Hematopoiesis from Pluripotent Stem Cells	0.030903
fMLP Signaling in Neutrophils	0.031623
Cell Cycle: G2/M DNA Damage Checkpoint Regulation	0.033884
Altered T Cell and B Cell Signaling in Rheumatoid Arthritis	0.038905
OX40 Signaling Pathway	0.040738
CCR3 Signaling in Eosinophils	0.040738
Cellular Effects of Sildenafil (Viagra)	0.040738
Reelin Signaling in Neurons	0.044668
Melanoma Signaling	0.045709
Systemic Lupus Erythematosus Signaling	0.046774
Estrogen-mediated S-phase Entry	0.046774
Prostate Cancer Signaling	0.047863

Supplementary Table 3. Upstream regulators and gene sets predicted to be activated differently following epithelial *Stat3* deletion in lungs of *Kras* mutant-induced female and male mice.

	Activation z-score	
**Upstream regulators**	**Female mice**	**Male mice**
HDAC1	2.59	-2.59
TCR	2.59	-1.98
HDAC2	2.57	-2.57
calcitriol	2.40	-2.40
SOX11	2.22	-2.22
MAP4K4	2.22	-2.22
BCL6	2.21	-2.21
miR-34a-5p (and other miRNAs w/seed GGCAGUG)	2.21	-2.21
GATA3	2.17	-2.17
GnRH analog	2.11	-2.11
2-amino-5-phosphonovaleric acid	1.98	-1.11
Ifnar	1.95	-1.95
Rock	1.73	-1.73
tretinoin	1.67	-2.55
IL15	1.55	-1.55
IL7	1.38	-1.38
ETS1	1.34	-1.34
miR-124-3p (and other miRNAs w/seed AAGGCAC)	1.30	-1.30
sphingosine-1-phosphate	1.26	-1.26
miR-27a-3p (and other miRNAs w/seed UCACAGU)	1.25	-1.25
SRF	1.23	-1.46
miR-17-5p (and other miRNAs w/seed AAAGUGC)	1.16	-1.16
let-7	1.10	-1.10
MIR17HG	1.07	-1.07
ATF3	1.07	-1.07
TCF3	1.04	-1.04
bexarotene	1.00	-1.34
ERBB3	1.00	-1.00
MKNK1	1.00	-1.00
NFATC2	1.00	-1.00
PRKAA1	1.00	-1.00
BNIP3L	1.00	-1.00
GH1	1.00	-1.00
mir-223	-1.00	1.34
WT1	-1.00	1.00
IKZF1	-1.03	1.03
PDGF BB	-1.04	1.81

CBX5	-1.07	1.07
GLI2	-1.07	1.07
FOXM1	-1.28	1.28
CCND1	-1.41	1.41
CREB1	-1.50	2.02
EZH2	-1.51	1.51
nocodazole	-1.65	1.65
PKD1	-1.71	1.00
mir-21	-1.72	1.72
troglitazone	-1.74	1.74
valproic acid	-1.75	1.09
RXRA	-1.87	1.87
clofibrate	-1.93	1.93
ATF4	-1.94	1.94
IGF1R	-1.95	1.95
COMMD3-BMI1	-2.00	2.00
geldanamycin	-2.00	1.34
phorbol myristate acetate	-2.34	2.97
benzo(a)pyrene	-2.41	1.00

Supplementary Table 4. List of primers used to analyze target immune genes by real-time PCR.

Gene	Forward	Reverse
Actin	5'GGCTGTATTCCCCTCCATCG3'	5'CCAGTTGGTAACAATGCCATGT3'
Cd45	5'ACCACCAGGTGAATGTCAATTT3'	5'CTTGCTTTCCCTCGGTTCTTT3'
Arg1	5'TTTTTCCAGCAGACCAGCTT3'	5'AGAGATTATCGGAGCGCCTT3'
Il6	5'CTGATGCTGGTGACAACCAC3'	5'CAGACTTGCCATTGCACAAC3'
Tnfa	5'GAACTGGCAGAGAAGAGGCAT3'	5'AGGGTCTGGGCCATAGAACT3'
Ido	5'TGGCGTATGTGTGGAACCG3'	5'CTCGCAGTAGGGAACAGCAA3'
Il17	5'CTCCAGAAGGCCCTCAGACTAC3'	5'GGGTCTTCATTGCGGTGG3'
Tgfb	5'CTCCCGTGGCTTCTAGTGC3'	5'GCCTTAGTTTGGACAGGATCTG3'
Ifng	5'GATGCATTCATGAGTATTGCCAAGT3'	5'GTGGACCACTCGGATGAGCTC3'
Ccl2	5'TTAAAAACCTGGATCGGAACCAA3'	5'GCATTAGCTTCAGATTTACGGGT3'
Foxp3	5'GGCCCTTCTCCAGGACAGA3'	5'GCTGATCATGGCTGGGTTGT3'
Tbx21	5'CAACAACCCCTTTGCCAAAG3'	5'TCCCCCAAGCAGTTGACA3'
Gzmb	5'CCACTCTCGACCCTACATGG3'	5' GGCCCCCAAAGTGACATTTATT3'
