# Supplementary material for: Sex specific function of epithelial STAT3 signaling in pathogenesis of K-ras mutant lung cancer
Source: Nat Commun. 2018 Nov 2;9:4589. doi: 10.1038/s41467-018-07042-y (PMC6214980; doi:10.1038/s41467-018-07042-y)
Supplement: Supplementary file 2 — Supplementary Data 1 [file 41467_2018_7042_MOESM2_ESM.pdf]

Reviewers' comments:

Reviewer #1:
(Remarks to the Author):

Caetano et al address the importance of STAT3 pathway in lung cancer. Surprisingly, they found that while STAT3 genetic ablation reduces tumors in females, it does increase lung tumorigenicity oin male mice. RNAseq analysis hints and some differentially regulated patwhays although how gender specific effect there works is not clear and it is not clear whether it is a cause of differences or a consequences of increased (males) or decreased (females) tumorigenicity in STAT3 knockout mice. Overal, the messeage of the paper is timely and very interesting, however more mechanistic insights would help and several issues remain to be addressed.

Major:
1) What is the mechanism (s) which link gender, STAT3 and expression of the microenviromental and immune genes described on Fig 2 and 3 which authors imply to be important for phenotypic differences?
2) Is not it counterintuitive that IL-6 blockade blocks tumorigenesis in male mice where STAT3 is already deleted? Does IL-6 act through STAT3?
3) Authors are correct that ER signaling is likely important for gender differences and was previously reported to control IL-6 synthesis. More pro-carcinogenic estrogen metabolite is found in tumors vs normal tissue, and, obviously in females vs males PMID: 29290988. Does tamoxifen block mostly some of the protective estrogen metabolites in this model?

Minor
1) Robert Eferl has recently published a Gastroenterology paper about gender-different phenotypes of STAT1 deletion in colon cancer. STAT1 and STAT3 antagonize each other transcription programs functionally. is it possible that gender-specific role of STAT1 and STAT3 is mediated by the same mechanisms- please discuss.

Reviewer #2:
(Remarks to the Author):

This paper describes a sex-differential role for Stat3 signalling in a K-ras mutant lung adenocarcinoma knockout mouse model. Stat3 deletion led to completely opposite effects in male and female mice, with increased anti-tumor responses in females and increased pro-tumor responses in males. A role for IL-6 in males and estrogen signaling in females was confirmed. This opens the potential for sex-differential targeted immunotherapy in this poor prognosis tumor. These findings are novel and add important information regarding these poor prognosis tumors.

In this study the authors are describing a "sex-specific" rather than "gender-specific" effect. Gender refers to different behavioural and societal factors in males and females, whereas this effect is linked to biological sex and sex hormones. The title should therefore be changed to reflect this as should the multiple other references to gender throughout the text. I would suggest using the words "sex-differential" rather than "sex-specific".

The paper would greatly benefit from a summary figure or table outlining what they think is going on the male and female tumor environment in terms of the role of estrogen, IL-6, KF-kB,STAT3, neutrophils and the other key anti- and pro-inflammatory factors affected.

Abstract
The abstract would benefit from a concluding sentence on the relevance of the findings.

Introduction

Line 60 – the words "understanding molecular underpinnings underlying…" is poor grammar and should be reworded.

Line 72-3 "STAT3, the IL-6-responsive transcription factor, activation…" likewise this sentence should be reworded to flow better since breaking up STAT3 and activation does not read well.

Line 76 – define CCSP

Line 77 – The STAT3 pathway.

Line 81 – The words "largely unknown" suggest something else other than the IL-6 role is known. Would it be better to state "otherwise unknown" or "largely unexplored"?

Results

Lines 97-102 This is a repetition of what has just been stated in the introduction.

Line 100 "underscored" is the wrong word to use here.

Line 101 – "remain" not "remained"

Line 122 "an average of"

Line 177 put "Il6" in italics

Lines 178-9 – The authors cannot categorically state that Th1 and CD8 T cells and NK cells were affected according to the increased expression of Ifng and Gzmb? IFN-γ can derive from any of those 3 populations and granzyme B can be produced by other cells too e.g Tregs. Suggest reword.

Line 207 – can the authors further expand on why this indicates a myeloid suppressor cell response?

Line 242 – Foxp3 and Ido are also markers of immune suppression and deserve mention here since both went up in the tamoxifen treated mice. There was also no mention of the marked rise in Il17 in treated mice which is likely to have contributed to the pro-inflammatory milieu.

Discussion

Line 265-6 – the authors cannot really say that NF-kB driven IL-6 expression is involved from their experiments. They simply showed an increase in NK-kB expression (p65 DNA binding in tumors) which might be expected in a tumor promoting environment and is not specific to IL-6 induction.

Regulatory T cells deserve a mention somewhere in the discussion. They play a key role in suppressing tumor immunity and the authors show effects on Foxp3 and Tgfβ, following anti-IL-6 treatment in males.

Line 324 – remove the comma after mention

Line 362 – "Th17 cell activity" not "cells"

Line 369 – define KRAS

Line 443 – "as done previously" not "as we previously done"

Figure 3 – for ease of interpretation it would be good if the authors could indicate the sex on the figure rather than just the legend.

Figure S3 should appear in the main text since it is integral to the conclusions.

Figure S4 needs to describe what the blue line shows.

Reviewer #3:
(Remarks to the Author):

The data presented showing differences in the response of male vs female mice in K-Ras dependent lung cancer with loss of Stat3 lung tumors are intriguing and potentially of high significance. The authors have performed multiple experiments defining changes in cytokines and immune populations which are likely to underlie the opposing effects on tumor progression. However, the studies have not defined a mechanism underlying this. The data showing the effects of tamoxifen are interesting but these are limited. Clearly there are a large number of changes in the setting of Stat3 deletion which are opposite in direction in male vs female. However, without more mechanistic insight, the manuscript as written is largely descriptive. The manuscript would have to develop at least some mechanistic insight as to how these opposing effects are occurring, and are they specific for Stat3 or do they reflect a more essential difference in how the TME is regulated in males v females.

POINT-BY-POINT RESPONSE

We thank the reviewers and the editor for their constructive comments and provide below a point-by-point response to the raised suggestions and concerns.

Editor's comments:

Comments: *"We would expect any revised manuscript to include tumour size (in addition to tumor number) as an indicator of tumour burden as suggested by Reviewer#3 in confidential comments to the editor. Percentage of lung that is tumor would also be fine as noted by Reviewer#3. However, despite the addition of further experimental evidence to support a molecular mechanism (as suggested by both Reviewer#1 and Reviewer#3) would greatly strengthen the manuscript we would not expect the mechanism to be fully sorted out."*

Response: We thank the editors for their constructive comments and overall positive comments. We now add data enumerating tumor areas in the different mice groups. As instructed, we have calculated percentages of lung occupied by tumors. These data are now included as a new supplementary figure (**Fig. S1**). We also now discuss these findings where applicable in the revised manuscript (**Methods section, page 20; Results section, page 5**).

Reviewer #1

General comment: *"Caetano et al address the importance of STAT3 pathway in lung cancer. Surprisingly, they found that while STAT3 genetic ablation reduces tumors in females, it does increase lung tumorigenicity oin male mice. RNAseq analysis hints and some differentially regulated patwhays although how gender specific effect there works is not clear and it is not clear whether it is a cause of differences or a consequences of increased (males) or decreased (females) tumorigenicity in STAT3 knockout mice. Overal, the messeage of the paper is timely and very interesting, however more mechanistic insights would help and several issues remain to be addressed."*

General response: We thank reviewer #1 for the overall positive feedback and provide responses to the concerns raised by the reviewer.

Comment 1: *"What is the mechanism (s) which link gender, STAT3 and expression of the microenviromental and immune genes described on Fig 2 and 3 which authors imply to be important for phenotypic differences?"*

Response 1: We apologize for inadvertently not clearly providing a plausible mechanism(s) that may underlie the disparate lung tumor phenotypes following *Stat3* deletion among the male and female mice. We further analyzed our RNA-sequencing (RNA-Seq) data topologically organizing the identified differentially modulated genes into topological functional gene-gene interaction networks with predicted activated or inhibited states. We found that estrogen signaling was predicted to be activated, following epithelial *Stat3* deletion, in female but not in male mice. Of note, we also found that gene networks comprising activated estrogen signaling/activation in female mice included elevated expression of infiltrating T cell markers (e.g. CD4), pointing to potential cues linking estrogen signaling activation and anti-tumor immune mechanisms. These data are now included as a new supplementary figure in the revised manuscript (**Fig. S4**) and discussed where applicable in the revised manuscript (**Results section, page 11; Discussion section, page 13**).

It is important to mention that we had found, in the original submitted manuscript, increased NF-κB activation, evidenced by increased p65 DNA binding activity, in tumors from male LR/*Stat3*$^{\Delta/\Delta}$ mice relative to male CC-LR littermates but not in female LR/*Stat3*$^{\Delta/\Delta}$ counterparts. These suggest that differential activation of NF-kB may underlie the observed sex-associated disparity in *K-ras* mutant lung oncogenesis among male and female mice. We suggest that lack of epithelial *Stat3* increases NF-kB activation which in turn drives the pro-tumor immunosuppressive microenvironment in male mice and leads to tumor promotion. Of note, our findings suggest that this mechanism does not occur in female mice as alluded to before, in part, due to estrogen receptor signaling activation resulting in an anti-tumor immune microenvironment. These plausible mechanisms are now schematically summarized as a new Figure (**Figure 7**) in the revised manuscript and discussed where applicable (**Discussions section, pages 12, 14 and 17**).

Comment 2: *"Is not it counterintuitive that IL-6 blockade blocks tumorigenesis in male mice where STAT3 is already deleted? Does IL-6 act through STAT3?"*

Response 2: We agree that it may first appear counterintuitive that targeting IL-6 blocks tumorigenesis in a STAT3-dependent manner in male mice where *Stat3* is already deleted. However, it is important to mention that in the mouse models we employed, *Stat3* was conditionally and selectively depleted only in airway epithelium. It is thus plausible and likely that the tumor stroma and immune microenvironment still comprises *Stat3* which may be functionally targeted by IL-6 blockade. This further supports the role of lung tumor microenvironment in sex-specific pathogenesis of *K-ras* mutant lung cancer. We had previously reported on the role of *Stat3* in the microenvironment in the pathogenesis of *K-ras* mutant lung cancer and we now allude to this in the revised manuscript (**Discussion section, page 14**).

Comment 3: *"Authors are correct that ER signaling is likely important for gender differences and was previously reported to control IL-6 synthesis. More pro-carcinogenic estrogen metabolite is found in tumors vs normal tissue, and, obviously in females vs males PMID: 29290988. Does tamoxifen block mostly some of the protective estrogen metabolites in this model?"*

Response 3: We thank the reviewer for this constructive comment. It is possible that tamoxifen blocks some of the protective estrogen metabolites in this model. We cannot ascertain this important supposition now and we believe comprehensive assessment of these metabolites may be outside the scope of our current manuscript/study. Yet, and as mentioned to the same reviewer (see Response 1), following further analysis of our RNA-Seq data probing for functionally modulated (computationally) gene networks), we found that estrogen signaling is predicted to be activated following epithelial *Stat3* deletion in females only and the same topological network comprised elevated expression of T cell associated genes. These gene network data are now included as a new supplementary figure in the revised manuscript (**Fig. S4**) and discussed where applicable in the revised manuscript (**Results section, page 11**). We also now discuss in the revised manuscript (**Discussion section, page 16**) the possibility that differential carcinogenic estrogen metabolites may exist following Stat3 deletion in males and females and that protective metabolites may be impacted by tamoxifen treatment.

Comment 4 (minor): *"Robert Eferl has recently published a Gastroenterology paper about gender-different phenotypes of STAT1 deletion in colon cancer. STAT1 and STAT3 antagonize each other transcription programs functionally. is it possible that gender-specific role of STAT1 and STAT3 is mediated by the same mechanisms- please discuss."*

Response 4: We thank the reviewer for bringing up this important published report. This study by Crncec and colleagues shows a male specific tumor suppressor activity for epithelial STAT1 in colorectal cancer through modulation of anti-tumor CD8 T cell response. This is partly consistent with our finding that shows a tumor suppressive function for epithelial Stat3 in male mice and modulation of anti-tumor T cell response in male mice with lack of epithelial *Stat3*. It is important to note that we have interrogated our gene expression data and found no evidence for modulation of Stat1 signaling in our models. We now incorporate and discuss the report by Eferl's group in the **Discussion section (page 15)** of the revised manuscript.

Reviewer #2

General comment: *"This paper describes a sex-differential role for Stat3 signalling in a K-ras mutant lung adenocarcinoma knockout mouse model. Stat3 deletion led to completely opposite effects in male and female mice, with increased anti-tumor responses in females and increased pro-tumor responses in males. A role for IL-6 in males and estrogen signaling in females was confirmed. This opens the potential for sex-differential targeted immunotherapy in this poor prognosis tumor. These findings are novel and add important information regarding these poor prognosis tumors."*

General response: We thank the reviewer for the positive feedback and for finding that our study is novel and important.

Comment 1: *"In this study the authors are describing a "sex-specific" rather than "gender-specific" effect. Gender refers to different behavioural and societal factors in males and females, whereas this effect is linked to biological sex and sex hormones. The title should therefore be changed to reflect this as should the multiple other references to gender throughout the text. I would suggest using the words "sex-differential" rather than "sex-specific"."*

Response 1: To comply with the reviewer's suggestion, we have changed the term "gender" to "sex" throughout the manuscript, and term "sex-specific" to "sex-differential" where applicable.

Comment 2: *"The paper would greatly benefit from a summary figure or table outlining what they think is going on the male and female tumor environment in terms of the role of estrogen, IL-6, KF-kB, STAT3, neutrophils and the other key anti- and pro-inflammatory factors affected."*

Response 2: A new figure is now included in the revised version of the manuscript (**Figure 7**) that schematically summarizes cues linking estrogen, IL-6, NF-kB, STAT3 and other pro- and anti-tumor immune mechanisms. This figure is also discussed in the Discussion section of the revised manuscript.

Comment 3: *"The abstract would benefit from a concluding sentence on the relevance of the findings."*

Response 3: A concluding sentence is now included in the revised version that suggests that immunotherapy against *K-ras* mutant lung cancer may be improved by personalized (e.g. sex-based) strategies.

Comment 4: *"Introduction: Line 60 – the words "understanding molecular underpinnings underlying…" is poor grammar and should be reworded."*

Response 4: This has been reworded to “understanding molecular underpinnings of this particular type of lung malignancy”.

Comment 5: *“Line 72-3 “STAT3, the IL-6-responsive transcription factor, activation…” likewise this sentence should be reworded to flow better since breaking up STAT3 and activation does not read well.”*

Response 5: This is now reworded to “Activation of STAT3, an IL-6-responsive transcription factor, was shown to…..”.

Comment 6: *“Line 76 – define CCSP”*

Response 6: We now defined CCSP (club cell secretory protein).

Comment 7: *“Line 77 – The STAT3 pathway.”*

Response 7: We now included “the” before STAT3 pathway.

Comment 8: *“Line 81 – The words “largely unknown” suggest something else other than the IL-6 role is known. Would it be better to state “otherwise unknown” or “largely unexplored”?”*

Response 8: We have replaced it with “largely unexplored” as suggested.

Comment 9: *“Results: Lines 97-102 This is a repetition of what has just been stated in the introduction.”*

Response 9: We originally included these phrases to accentuate our *K-ras* mutant lung cancer model and previous findings. To comply with the reviewer’s suggestion, we now reduced these phrases and the repetition.

Comment 10: *“Line 100 “underscored” is the wrong word to use here.”*

Response 10: Since we reduced the accompanying phrases we eliminated the term “underscored”.

Comment 11: *“Line 101 – “remain” not “remained””*

Response 11: This was corrected.

Comment 12: *“Line 122 “an average of””*

Response 12: This was corrected.

Comment 13: *“Line 177 put “Il6” in italics”*

Response 13: *Il6* was italicized.

Comment 14: *“Lines 178-9 – The authors cannot categorically state that Th1 and CD8 T cells and NK cells were affected according to the increased expression of Ifng and Gzmb? IFN-γcan derive from*

any of those 3 populations and granzyme B can be produced by other cells too e.g Tregs. Suggest reword."

Response 14: To comply with the reviewer's suggestion we removed suppositions on the immune cellular source of Ifn-g and Gzmb.

Comment 15: *"Line 207 – can the authors further expand on why this indicates a myeloid suppressor cell response?"*

Response 15: We removed the discussion on the potential implication of a suppressive myeloid cell response in the Results section of the revised manuscript.

Comment 16: *"Line 242 – Foxp3 and Ido are also markers of immune suppression and deserve mention here since both went up in the tamoxifen treated mice. There was also no mention of the marked rise in Il17 in treated mice which is likely to have contributed to the pro-inflammatory milieu."*

Response 16: We thank the reviewer for raising this important point. To comply with the reviewer's suggestion, we noted in the applicable section of the revised manuscript (**Results section, page 11**) the elevated expression of *Foxp3*, *Ido* and *Il17* – suggestive of immune suppression and protumor immune response – in lungs of tamoxifen treated female LR/*Stat3*$^{\Delta/\Delta}$ mice.

Comment 17: *"Discussion: Line 265-6 – the authors cannot really say that NF-kB driven IL-6 expression is involved from their experiments. They simply showed an increase in NK-kB expression (p65 DNA binding in tumors) which might be expected in a tumor promoting environment and is not specific to IL-6 induction."*

Response 17: We agree with the reviewer that the elevated activation of NF-κB in our phenotype is perhaps associative rather than causal of elevated *Il6* expression. It is important to note that we had assessed DNA binding activity of p65 as a surrogate of NF-κB activity. Additionally, we build on previous reported work including our own that had shown the crucial role for NF-κB in transactivation and expression of *Il6*. To comply with the reviewer's suggestion, we have toned down our conclusions with respect to the causal link between NF-κB activation and *Il6* expression where applicable in the revised version of the manuscript. In addition, the newly incorporated mechanistic/schematic summary figure (**Figure 7**) in the revised manuscript includes dashed, rather than solid, lines when discussing the plausible roles (not fully determined in the present study) for NF-κB in these models.

Comment 18: *"Regulatory T cells deserve a mention somewhere in the discussion. They play a key role in suppressing tumor immunity and the authors show effects on Foxp3 and Tgfβ, following anti-IL-6 treatment in males."*

Response 18: We thank the reviewer for this important comment. We now discuss the role of regulatory T cells in the revised version of the manuscript (**Discussion section, page 13**).

Comment 19: *"Line 324 – remove the comma after mention"*

Response 19: The comma was removed.

Comment 20: *"Line 362 – "Th17 cell activity" not "cells""*

Response 20: This was corrected.

Comment 21: *"Line 369 – define KRAS"*

Response 21: We had defined KRAS (Kirsten rat sarcoma viral oncogene) previously in the manuscript.

Comment 22: *"Line 443 – "as done previously" not "as we previously done""*

Response 22: This was corrected.

Comment 23: *"Figure 3 – for ease of interpretation it would be good if the authors could indicate the sex on the figure rather than just the legend."*

Response 23: To comply with the reviewer's suggestion, the sex of mice was added to the figures in the revised manuscript.

Comment 24: *"Figure S3 should appear in the main text since it is integral to the conclusions."*

Response 24: **Figure S3** was now added to the original manuscript as **Figure 4**.

Comment 25: *"Figure S4 needs to describe what the blue line shows."*

Response 25: This is now done for the previous Figure. S4 (**now Figure. S5**).

Reviewer #3

General comment: *"The data presented showing differences in the response of male vs female mice in K-Ras dependent lung cancer with loss of Stat3 lung tumors are intriguing and potentially of high significance. The authors have performed multiple experiments defining changes in cytokines and immune populations which are likely to underlie the opposing effects on tumor progression. However, the studies have not defined a mechanism underlying this. The data showing the effects of tamoxifen are interesting but these are limited. Clearly there are a large number of changes in the setting of Stat3 deletion which are opposite in direction in male vs female. However, without more mechanistic insight, the manuscript as written is largely descriptive. The manuscript would have to develop at least some mechanistic insight as to how these opposing effects are occurring, and are they specific for Stat3 or do they reflect a more essential difference in how the TME is regulated in males v females."*

General response:

We thank the reviewer for the constructive comments. As mentioned earlier in this point-by-point response, we apologize for inadvertently not clearly providing a plausible mechanism(s) that may underlie the disparate lung tumor phenotypes following *Stat3* deletion among the male and female mice. We further analyzed our RNA-Seq gene expression data topologically to organize the identified differentially modulated genes into topological gene networks with predicted functionally modulated (activated or inhibited) states. This analysis demonstrated that estrogen signaling was predicted to be activated, following epithelial *Stat3* deletion, in female but not in male mice. Of note, we also found

that gene networks comprising activated estrogen signaling activation in female mice included elevated expression of infiltrating T cell markers (e.g. CD4), pointing to potential cues linking estrogen signaling activation and anti-tumor immune mechanisms. These data are now included as a new supplementary figure in the revised manuscript (**Fig. S4**) and discussed where applicable in the revised manuscript (**Results section, page 11; Discussion section, pages 12 and 14**).

It is noteworthy that we had already provided some mechanistic insight, namely: interaction between estrogen signaling and NF-kB associated immune responses examined by targeting ER using tamoxifen in female mice, and IL-6 blockade and neutrophil depletion in male mice, respectively). It is important to mention that we had found, in the original submitted manuscript, increased NF-κB activation, evidenced by increased p65 DNA binding activity, in tumors from male LR/*Stat3*$^{\Delta/\Delta}$ mice relative to male CC-LR littermates but not in female LR/*Stat3*$^{\Delta/\Delta}$ counterparts. These suggest that differential activation of NF-kB may underlie the observed sex-associated disparity in *K-ras* mutant lung oncogenesis among male and female mice. We suggest that lack of epithelial *Stat3* increases NF-kB activation which in turn drives the pro-tumor immunosuppressive microenvironment in male mice and leads to tumor promotion. Of note, our findings suggest that this mechanism does not occur in female mice as alluded to before, in part, due to estrogen receptor signaling activation resulting in an anti-tumor immune microenvironment. These plausible mechanisms are now schematically summarized as a new Figure (**Figure 7**) in the revised manuscript and discussed where applicable (**Discussions section, pages 12, 14 and 17**).

REVIEWERS' COMMENTS:

Reviewer #1 (Remarks to the Author):

authors sufficiently addressed the comments

Reviewer #2 (Remarks to the Author):

I am satisfied with the revised manuscript and answers to my questions

Reviewer #3 (Remarks to the Author):

The authors have addressed comments from the previous review. In particular they have elaborated on a potential mechanism underlying the differential response of male and female mice in the setting of STAT3 loss. This is clearly summarized in the new Fig. 7 which clarifies the data presented. In addition they have addressed the comments from all of the reviewers and revised the text as well as adding new figures. Overall the revised manuscript is significantly improved and presents important data defining sex specific differences in the response of the mice leading to opposing effects on tumor progression.
