# Supplementary material for: Sex specific function of epithelial STAT3 signaling in pathogenesis of K-ras mutant lung cancer
Source: Nat Commun. 2018 Nov 2;9:4589. doi: 10.1038/s41467-018-07042-y (PMC6214980; doi:10.1038/s41467-018-07042-y)
Supplement: Supplementary file 5 — Peer Review File [file 41467_2018_7042_MOESM5_ESM.pdf]

Description of Additional Supplementary Files

File Name: Supplementary Data 1
Description: Gene features modulated differently following epithelial Stat3 deletion in lungs of Kras mutant-induced female and male mice.

File Name: Supplementary Data 2
Description: Diseases and biological functions predicted to be modulated differently following epithelial Stat3 deletion in lungs of Kras mutant-induced female and male mice.
